# Is Delirium the Cognitive Harbinger of Frailty in Older Adults? A Review about the Existing Evidence

**DOI:** 10.3389/fmed.2017.00188

**Published:** 2017-11-08

**Authors:** Giuseppe Bellelli, Rosamaria Moresco, Paola Panina-Bordignon, Beatrice Arosio, Cecilia Gelfi, Alessandro Morandi, Matteo Cesari

**Affiliations:** ^1^Geriatric Unit, San Gerardo Hospital, Monza, Italy; ^2^School of Medicine and Surgery, University Milano-Bicocca, Milan, Italy; ^3^National Research Council (CNR), Nuclear Medicine Department, San Raffaele Hospital (IRCCS), Milan, Italy; ^4^San Raffaele Hospital (IRCCS), Milan, Italy; ^5^Geriatric Unit, Department of Medical Sciences and Community Health, University of Milano, Fondazione IRCCS Ca’ Granda, Ospedale Maggiore Policlinico, Milan, Italy; ^6^Department of Biomedical Sciences for Health, University of Milano, Segrate, Italy; ^7^Department of Rehabilitation and Aged Care, Casa di Cura “Ancelle della Carità”, Fondazione Teresa Camplani, Cremona, Italy; ^8^Geriatric Unit, Fondazione IRCCS Ca ‘Granda, Ospedale Maggiore Policlinico, Milan, Italy

**Keywords:** frailty, delirium, older adults, review of literature pathophysiology, geriatric syndromes

## Abstract

Frailty is a clinical syndrome defined by the age-related depletion of the individual’s homeostatic reserves, determining an increased susceptibility to stressors and disproportionate exposure to negative health changes. The physiological systems that are involved in the determination of frailty are mutually interrelated, so that when decline starts in a given system, implications may also regard the other systems. Indeed, it has been shown that the number of abnormal systems is more predictive of frailty than those of the abnormalities in any particular system. Delirium is a transient neurocognitive disorder, characterized by an acute onset and fluctuating course, inattention, cognitive dysfunction, and behavioral abnormalities, that complicates one out of five hospital admissions. Delirium is independently associated with the same negative outcomes of frailty and, like frailty, its pathogenesis is usually multifactorial, depending on complex inter-relationships between predisposing and precipitating factors. By definition, a somatic cause should be identified, or at least suspected, to diagnose delirium. Delirium and frailty potentially share multiple pathophysiologic mechanisms and pathways, meaning that they could be thought of as the two sides to the same coin. This review aims at summarizing the existing evidence, referring both to human and animal models, to postulate that delirium may represent the cognitive harbinger of a state of frailty in older persons experiencing an acute clinical event.

## Introduction

Although frailty and delirium are intuitively associated, a clear taxonomy of their biological and clinical relationship has not provided yet in geriatric medicine.

For many years, age has been considered as one of the most powerful predictors of mortality and adverse outcomes in older people. However, growing empirical evidence and several scientific publications have clearly shown that “chronological age” is not able *per se* to capture with sufficient accuracy the extreme heterogeneity of the health status in older persons (1, 2). In order to promote a measure more focused on the individual’s functions and biology, the concept of frailty has received special attention over the past years. In fact, frailty has been indicated as a condition which may accurately capture the homeostatic reserves of the organism and, as such, improve the assessment of the risk profile. In other words, frailty might represent the new criterion for defining the individual as (biologically) old and replace the obsolete age concept (2). Interestingly, this change of paradigm might also support a more person-tailored approach in the design of clinical interventions.

According to a commonly accepted definition, frailty is defined as a medical syndrome characterized by a decrease of functional reserve capacities, diminished strength, and endurance. The consequence of this increased vulnerability is that a frail person is more prone than a non-frail to develop negative health-related outcomes, including decline in functional and motor performance, prolonged length of hospital stay, institutionalization, rehospitalization, and mortality (2–4). Frailty might thus be considered as the complex biological background on which multiple protective and disruptive factors interact in the determination of the clinical manifestations and negative outcomes (2, 4). From a pathophysiological perspective, it is well accepted that the physiological systems which are involved in the determination of frailty, including brain, endocrine system, immune system, and skeletal muscle, are mutually interrelated, so that when decline starts in a given system, implications may also regard the others. To support this, it has shown that the number of abnormal systems is more predictive of frailty than are the abnormalities in any particular system (4). Recently, to explore the mechanistic relationship between aging, frailty, and mortality, Rutenberg et al. developed a computational model in which possible health attributes are represented by the nodes of a complex network, with the connection showing a scale-free distribution (1). Each node can be either damaged (i.e., a deficit) or undamaged. The most connected nodes are the mortality nodes; the next most connected nodes are frailty nodes that broadly correspond to clinically or biologically significant health characteristics. According to this model, individuals die when mortality nodes are highly damaged. Nodes are damaged randomly reflecting environmental influences, intrinsic features, and their interactions (5). Through interactions, the rate of damage of an individual node increases as more of its connected neighbors are damaged. This model can explain why frail individuals are at higher risk of vulnerability and mortality than robust ones, and facilitates the initial understanding of the factors influencing the health trajectories of older individuals (1).

Delirium is a transient neurocognitive disorder, characterized by an acute onset and fluctuating course, inattention, cognitive dysfunction, and behavioral abnormalities, which develops in association with another underlying medical condition (6). Sometimes, though not invariably, delirium presents with behavioral disturbances, including sleep-wake cycle disruption, psychotic symptoms, and agitation (7). It has been shown that delirium complicates about one out of five hospital admissions (8, 9), representing a clear burden for the patient as well as for public health. Like frailty, delirium is independently associated with a number of negative outcomes, including increased length of hospital stay, elevated healthcare costs, accelerated cognitive impairment, delayed or limited recovery of functional decline, increased risk of institutionalization, and mortality (10–14). In addition, delirium may cause patient and caregiver’s emotional distress (15, 16). Although a single factor can cause it (e.g., infections), its pathogenesis is usually multifactorial (10), depending on complex inter-relationships between predisposing and precipitating factors acting on the substratum of biological vulnerability (i.e., frailty). According to this view, delirium can thus be regarded as a clinical consequence of frailty in older persons experiencing stressful events. It is also important to mention that frailty and delirium are expected to rise in their prevalence in the next years, largely due to global aging of the populations worldwide.

In this review, we will summarize the existing evidence on the relationship between the two conditions (i.e., frailty and delirium), referring both to human and animal models.

## Commonalities and Differences Between Frailty and Delirium

Frailty and delirium share several commonalities but also have specific differences (Table 1). Both should be considered as multifactorial health conditions, characterized by multiple risk factors and causation which are not necessarily specific to a given organ system failure. This notion is indirectly confirmed by a growing body of evidence, from cardiology (17, 18) to infectious disease medicine (19, 20), from oncology (21, 22) to anesthesiology (23, 24), that these two conditions have a crucial role in clinical and research areas. Both frailty and delirium share many commonalities. In particular, they are both predictive of several negative health-related outcomes, most of which might be prevented by applying adapted and personalized interventions. A common biological substratum between the two conditions can also be suggested, possibly involving inflammation, endocrine and vascular systems, and oxidative stress (25). However, since both frailty and delirium find their biological roots in the aging process, it might be hypothesized that the same mechanisms responsible for the aging of the individual may become the causes of the conditions of interest when abnormally enhanced/stimulated by negative (endogenous or exogenous) stressors.

**Table 1:** Commonalities and differences between delirium and frailty.

Criteria	Delirium	Frailty
Definition	Neuropsychiatric syndrome characterized by acute and fluctuating deterioration in cognition, which develops in association with underlying medical conditions	Long-term clinical condition characterized by decrease of functional reserves, increasing vulnerability towards endogenous/exogenous stressors

Features	Inattention, thought disorders, impaired arousal, and behavioral abnormalities	Reduced homeostatic reserves due to age-related accumulation of deficits. Major physical features are characterized by malnutrition, abnormal energy expenditure, mobility impairment, and weakness

Prevalence	Delirium occurs in one in five hospitalized patients. Although less frequently, it can also occur in patients at home. Its prevalence is expected to rise in next years, due to the progressive ageing of population	About 10% of older community-dwellers have frailty, rising to between a quarter and a half of those aged over 85 years. The prevalence of frailty is expected to rise in next years, due to the progressive aging of the population

Time course	Acute onset (hours or days) with fluctuation in severity and duration; most cases are transient, resolving after a few days, but some persist for weeks or months	Chronic; in most cases, it is a progressive and irreversible disorder if adequate interventions are not applied

Pathophysiology	Inflamm-aging and immune-senescence are prerequisite for its onset. Hypothesized pathophysiologic mechanisms include inflammation, oxidative stress, neuroendocrine dysfunction, and circadian dysregulation	Inflamm-aging, immune-senescence, and endocrine dysfunction represent the cornerstones for the frailty biology

Impact on cognitive domain	Delirium is a strong predictor of new-onset dementia and acceleration of existing cognitive decline	Frailty, even when considered as a mere physical condition, is capable of substantially affect cognitive function. A bidirectional relationship between frailty and cognitive impairment has been demonstrated

Impact on functional domain	Delirium may affect mobility, especially in patients with increased pre-delirium vulnerability. It can also affect long-term functional performances	After exposure to endogenous/exogenous stressors, frailty may negatively affect the capacity to recover and regain or maintain functional independence

At the same time, frailty and delirium also differ in many aspects. Frailty is the long-term result of a decline in the homeostatic individual’s capacity across multiple physiological systems and it is usually considered as the endpoint of the progressive activity of corrosion exerted by chronic diseases during the normal aging process. On the contrary, delirium is an acute condition that occurs in response to a stressor (generally a medical problem) that may have a relatively rapid resolution, though sometimes it can persist weeks or even months. Delirium can be thought as an acute brain failure, reflecting the interaction between a predisposing factor (i.e., brain vulnerability) and one or more precipitating factor (i.e., the noxious insults), in which the brain is not able to compensate. Frailty may thus represent the ideal pabulum for the development of delirium, and delirium, on its side, may represent the clinical manifestation of underlying frailty in a patient suffering from an acute decompensation.

The relationship between frailty and delirium is even more complicated than above depicted. From a clinical perspective, frailty cannot be considered exclusively a pure disorder of function, though the criteria that are currently used for its definition may suggest. Indeed, there is empirical evidence that isolating physical from cognitive performance is really challenging in several cases. Some researchers have even proposed the terms of “cognitive frailty” and “reversible cognitive frailty” to describe heterogeneous cognitive conditions characterized by the simultaneous presence of both physical frailty and cognitive impairment (26). It is noteworthy that these concepts nest the idea of a reversible condition, the characteristic of dynamic mechanisms linking the physical and cognitive domains. The demonstration that cognitive impairment might reverse over time has been provided by a recent systematic review, showing that mild cognitive impairment (MCI) can return to normality with 8% reversion rate in clinical-based studies and 25% rate in population-based studies. The frequency of reversion from MCI to normality further increases to 26% when considering only studies of better quality (27).

Similar to what occurs for frailty, even delirium cannot be regarded only as an isolated mental disorder but there is evidence that it affects motor function as well. A study by Bellelli et al. compared four groups of 15 patients [with delirium alone, with dementia alone, with delirium superimposed on dementia (DSD), and with neither delirium nor dementia], finding that the mechanisms leading to the onset of delirium can also worsen motor performance (11). Other studies indirectly support such hypothesis, showing that delirium can complicate the functional recovery after adverse clinical events (13, 28). The reasons underlying this phenomenon are under study. It is possible that delirium causes motor fluctuations due to the disrupture of key central neurotransmitters (for example, related to attentive and executive functions) leading to an inability in planning and sustaining movement (11). According to Rockwood, it can also be hypothesized that the mobility impairment accompanying delirium is a reflection of the whole-system failure. Indeed, when complex systems collapse, their failure follows a cascade where highest order functions decline first. As such, the mobility impairment occurring in the course of delirium may represent a sign of a complex system close to failure. The more critical the individual’s health status is before the delirium onset, the higher will then be the likelihood that delirium will lead to mobility impairment (29).

Under a broad viewpoint, frailty reflects the life-long accumulation of deficits, thus defining the more or less state of vulnerability of the individual. Such (more or less overt) accentuated susceptibility to stressors represents the biological background where delirium might find its onset. In an optimal scenario, frailty should be detected in order to take adequate preventive countermeasures for avoiding the onset of its negative outcomes (including delirium). Nevertheless, delirium might become the condition making clinically evident for the first time a previously unknown/overlooked substratum of frailty.

## Review of Studies on Frailty and Delirium in Humans

To date, only few studies have specifically focused on the relationship between frailty and delirium in older people, and even less have assessed if frailty is a predictor of delirium. In a prospective observational study in 133 elective cardiac surgery patients, frailty was assessed using three different methods, i.e., a Modified Fried Criteria (MFC), the Short Physical Performance Battery (SPPB), and a 35-itemFrailty Index (FI). The primary exposure variable was postoperative delirium, assessed using the Confusion Assessment Method (CAM) (30). A proportion of patients ranging from 35.3 to 66.2% were frail, according to the method used to define it, and 18% had postoperative delirium. After adjusting for covariates, the presence of frailty resulted in a threefold to eightfold increase in risk of postoperative delirium, independent of the severity of the cardiac disease. In another study (31), carried out in 89 patients who underwent trans-catheter aortic valve implantation (TAVI), frailty was assessed by clinical judgment and as a summary score from baseline components, including the score assigned for Mini–Mental State Examination; Basic Activities of Daily Living; Instrumental Activities of Daily Living; Mini Nutritional Assessment, and impaired mobility. Delirium was assessed according to the Diagnostic and Statistical Manual of Mental Disorders, Fourth Edition (DSM-IV). Again, frailty predicted delirium onset and conferred additional value in the prediction of mortality after TAVI but only when frailty was assessed by subjective clinical judgment. On the contrary, such association was not found when frailty was assessed using the summary score. A third small study of older non-cardiac surgical patients evaluated whether a preoperative frailty score was an independent predictor of postoperative delirium. One-third of patients were frail and 25% developed postoperative delirium. In the multivariable logistic regression, frailty score (odds ratio = 1.84; 95% confidence interval = 1.07–3.1; *P* = 0.028) was independently associated with the development of postoperative delirium. More recently, in a prospective cohort of older patients admitted to a specialized delirium unit, Chew et al. assessed frailty with a 20-item index derived using items from a comprehensive geriatric assessment and delirium using the CAM (32). The authors also measured the residual sub-syndromal delirium (RSSD) before discharge from the unit by using the Delirium Rating Scale-Revised-98 severity score. The functional status was measured the modified Barthel Index on admission and 12 months post-delirium. In a logistic regression model, independent predictors of RSSD were as follows: frailty (OR 4.1, 95% CI 2.1–8.2, *P* < 0.001), the severity of delirium symptoms on admission (OR 1.2, 95% CI 1.1–1.2), and a pre-existing dementia (OR 4.2, 95% CI 2.0–8.6) (32). Interestingly, RSSD significantly mediated the effect of baseline frailty status on functional recovery at 12 months (32).

Other studies have assessed whether the coexistence of frailty and delirium is associated with an increased risk of death (33), or if delirium was associated with higher levels of frailty, in both studies finding that it was the case (34). However, other studies did not find a significant relationship between these two conditions (35–37).

Differences in the methods used to assess frailty and delirium as well as the selected populations and the length of follow-up can explain the heterogeneity in the study results. Taken together, the data from these studies suggest that further research is urgently needed to understand the complex relationship between frailty and delirium.

A further point is whether delirium may predispose itself to frailty. Indeed, several studies have demonstrated that delirium may be a risk factor for not only for dementia or a worsening of a preexisting dementia (14, 38), but also for subsequent functional impairment (12, 13, 39). Patients with persistent delirium are also less likely to regain activity of daily living function in comparison with non-delirious patients (40, 41). Moreover, when delirium is superimposed on dementia (which may represent itself a marker of pre-existing frailty), the risk to die in the middle short term is overall increased (42). It can be therefore hypothesized that the persistent or residual effects of delirium may delay or even hamper cognitive and functional recovery, ultimately resulting in new or increasing frailty and long-term disability and institutionalization (25). Future studies should better clarify this point.

## Common Biomarkers and Pathophysiological Mechanisms of Frailty and Delirium

A premise is required before describing the pathophysiological mechanisms proposed for both frailty and delirium. With aging, a number of changes occur in several interrelated physiological systems, one of the most important being the immune system. The changes in the immune system that occurs with aging are termed “immunosenescence” and may be defined as an age-associated decline in immune function that includes increased susceptibility to infections, reduced vaccination responses, and increased risk of chronic inflammatory diseases. Immunosenescence occurs in parallel with inflamm-aging, i.e., the increased presence of a low-grade chronic systemic inflammatory state typical of older age (43, 44). Inflamm-aging is characterized by increased levels of proinflammatory cytokines [e.g., interleukin (IL)-1β, IL-6, tumor necrosis factor-α (TNF-α), C-reactive protein (CRP)] and reduced concentrations of anti-inflammatory cytokines (e.g., IL-10, IL-1RA) (43). A variety of tissues (e.g., adipose tissue, muscle), organs (e.g., brain, liver), systems (e.g., immune system), and ecosystems (e.g., gut microbiota) of the body may contribute in a different manner to the onset and progression of inflamm-aging (45). Immunosenescence and inflamm-aging are particularly relevant for the pathophysiology of both delirium and frailty.

The exact pathophysiological mechanisms of delirium are not completely understood. A recent review by Maldonado suggests that at least five mechanisms are involved in delirium pathophysiology, including neuronal aging, neuroinflammation, oxidative stress, neuroendocrine dysregulation, and circadian dysregulation (46). In this review, we will focus exclusively on the three mechanisms which are thought to be more relevant for a common understanding of both delirium and frailty pathophysiology.

The neuroinflammatory hypothesis of delirium proposes that an acute peripheral inflammatory trigger (either infective, surgical, or traumatic) can provoke the activation of brain parenchymal cells, leading to an overexpression of proinflammatory cytokines and inflammatory mediators in the brain parenchyma, neuronal, and synaptic dysfunction and subsequent cognitive and behavioral symptoms of delirium (47). Importantly, brain is able to constantly monitor the presence of peripheral inflammation and how, upon exposure to specific stimuli, individuals may react to illness with a “sickness behavior,” i.e., a constellation of non-specific physiologic and behavioral signs and symptoms, including fever, malaise, fatigue, anorexia, lethargy, social withdrawal, and depressed mood (48, 49). According to the neuroinflammatory hypothesis, delirium may thus represent the CNS manifestation of a systemic disease, with an overproduction of cytokines that provoke a chain reaction in the neuronal cells of the brain (47). The immune signals and cytokines may enter the brain through two pathways (i.e., the neural pathway and the humoral pathway) where they determine the release of other proinflammatory cytokines by macrophage-like cells expressing toll-like receptors. In the neural pathway, the cytokines may activate primary afferent nerves, such as the vagus, and enter the brain through saturable transport system. In the humoral pathway, cytokines enter the brain at the level of the choroid plexus and the circumventricular organs. When enter the brain, cytokines may activate microglial cells. Microglial cells are the resident macrophages of the CNS and represent the 5–10% of all CNS cells. In the healthy brain, these are in a quiescent state, but, when they detect injured CNS cells or invading pathogens, they are able to adopt a specific phenotype with an amoeboid morphology. Phenotype modifications lead to further secretion of proinflammatory cytokines, and the expression of different cell surface receptors. With immune-senescence, microglial cells are characterized by an exaggerated proinflammatory response, acquiring a phenotype of primed less ramification, and reducing their chemotactic, phagocytic, and regulatory functions. A primed microglial phenotype is present also in chronic neurodegenerative processes where microglia cells lost their supportive role in neuroplasticity, thus favoring cognitive decline and synaptic dysfunction (50–53). Several studies, in patients of surgical and medical hospital wards, have shown that delirium is associated with significantly higher circulating levels of these inflammatory markers in comparison with non-delirium (54, 55). Importantly, cytokines can disrupt the neurotransmitter system balance, leading to reduced acetylcholine release (56) and decreased cholinesterase activity (57) and can activate the microglial cells, provoking an inflammatory response which can interfere with the connection and transmission functions of synapses (58, 59).

The oxidative stress hypothesis proposes that a number of physiologic and pathological events, such as tissue damage, hypoxia, illness, and infections, may lead to increased oxygen consumption, decreased oxygen availability, and reduced cerebral oxidative metabolism, which in turn may provoke cerebral dysfunction and associated cognitive and behavioral symptoms of delirium (47). Abnormal oxidative stress has been found in patients undergoing cardiopulmonary bypass surgery and in intensive care unit patients (60, 61). In one of these studies, Seaman has also shown that poor oxygenation is associated with cerebral dysfunction. Among a cohort of 101 patients, the authors assessed three measures of oxygenation (hemoglobin, hematocrit, pulse oximetry) and two measures of oxidative stress (sepsis, pneumonia), finding that indicators of oxidative dysfunction were more common in those who developed more frequently delirium, and this was not linked to illness severity (60). Pericytes may also be a potential target of interest in this framework. The pericytes are specific cells located at the abluminal side of the brain and muscular capillaries, which have the potential to express the inducible nitric oxide synthase (iNOS) and generate reactive oxygen and nitrogen species (RONS) (62). These studies suggest that pericytes, under specific circumstances such as an increased inflammatory status, may not only increase the production of iNOS and RONS but also by behaving as immune cells they are able to enhance the inflammatory response (63). Taken together, these data suggest that an increased oxidative stress at vascular level and in the brain parenchyma may predispose and underlie delirium development, with potential interaction between inflammation and oxidative stress.

The neuroendocrine hypothesis proposes that delirium reflects a reaction to acute stress. It is commonly accepted that stress can activate the hypothalamic–pituitary–adrenal axis: stressors activate the paraventricular nucleus of the hypothalamus resulting in the release of corticotrophin-releasing hormone, which acts on the pituitary gland, releasing adrenocorticotrophic hormone, which promotes glucorticoid release from the adrenal gland (47). Under normal circumstances, glucorticoids act to help the body in coping with the demands imposed by stress exposure, but there is evidence demonstrating that glucorticoids secreted during stress can have deleterious effects in the brain, inducing delirium and cognitive dysfunction (64, 65). Current evidence also suggests that the highly catabolic glucorticoids induce a general metabolic vulnerability in brain neurons and thus compromise their ability to survive various toxic insults (66), indirectly suggesting that the effect of increased glucorticoid secretion may be not always reversible.

The pathophysiology of frailty is complex too. With aging, the muscle undergoes several changes in its structure and composition, which are in part related to both immunosenescence and inflamm-aging. For example, proteomic studies in senescent mice have reported an increase of iron load and changes in redox homeostasis, associated with a severe loss of muscle function and loss of satellite cell recruitment (67). Importantly, these changes appear to occur in parallel with biochemical, morphological, and functional changes including a decrease of myelinated and unmyelinated fibers, ballooning, splitting, and enfolding of myelinated fibers (68) and decreased axonal neurofilaments (69). Other studies have confirmed that metabolic and structural changes are common between muscle and nerve, suggesting that both tissues may share a common signaling associated with muscle and nerve decline (67, 70). Furthermore, a release of metabolites, amino acids, and a dysregulation of myokine signaling seem to be related to “inflamm-aging” (44) with increased cytokine release.

The imbalance in the cytokine network may influence frailty either directly by promoting protein degradation or indirectly by affecting important metabolic pathways (71). A recent meta-analysis including 32 cross-sectional studies and 23,910 older subjects has shown that frailty and pre-frailty (i.e., a condition which is thought to be intermediate between the normal and the frailty status) are associated with significantly higher than normal serum inflammatory parameters, including CRP, IL-6, white blood cell, and fibrinogen levels (72). In other studies, frailty was associated also with lower serum levels of IL-12 and IL-23, two interleukins that are able to modulate the production of other interleukins (i.e., IL-17 and IL-22) in lymphocytes as well as the rapid recruitment of neutrophils in stressful conditions (73). Importantly, these changes in inflammatory patterns are consistent in frail individuals across various geographical regions and are associated with a decreased muscle strength, resistance to physical exercise and walking distance as determined by the 6-min walking test (74).

Cytokines dysregulation is also related to the lack of response to some hormones and anabolic factors (75), which typically underlie frailty development (75). These hormones include the dehydroepiandrosterone sulfate (DHEA-S), testosterone, cortisol, and insulin-like growth factor-1 (IGF-1). The DHEA, in particular, plays an important role in the maintenance of muscle mass (76) and both increased cortisol serum levels and an increased cortisol: DHEA-S ratios in the serum are associated with a decline in individual’s functional performance (77). Interestingly, the cortisol serum levels are elevated in frail individuals as compared with non-frail individuals, and are associated with both an increased muscle breakdown rate (78) and loss of bone density (79). The same role seems to be exerted by IGF-1 that is related to the maintenance of muscle mass structure and muscle strength by the way to inhibit apoptosis and to lower the oxidative stress in muscle (80).

In addition to the above-depicted mechanisms, it has recently been shown that body composition might play a dual role as source of inflammatory stimulus (through endocrine secretion of pro-inflammatory adipokines) (81) and target of the negative effects (i.e., induction of catabolic, apoptosis, autophagic muscular pathways) (82). This is of particular importance since sarcopenia (i.e., a loss of muscle mass and strength and/or reduced physical performance) is a key component of frailty, if not its central biological substrate (83). In this context, IL-6 was identified as being produced by immune cells as well as by muscle and adipose tissue, as also suggested by the fact that its expression acutely increases during muscle contraction. In addition, IL-6 induces insulin resistance which suppresses activity of various intrinsic and extrinsic modulators of muscle synthesis (84). Another pro-sarcopenic effect of inflammation is related to the generation of cortisol within tissues. Cortisol is profoundly catabolic and can be synthesized from inactive cortisone by the actions of the enzyme 11β-Hydroxysteroid dehydrogenase type 1 (11β-HSD1). The activity of the (11β-HSD1) increases with age and is induced by cytokines, including TNF-α and IL-6. Interestingly, IL-6 has been indicated as a cytokine for gerontologists due to its contribution in the pathogenesis in multiple age-related conditions (83).

## Review of Studies on Frailty and Delirium in Animals

**Figure 1:**
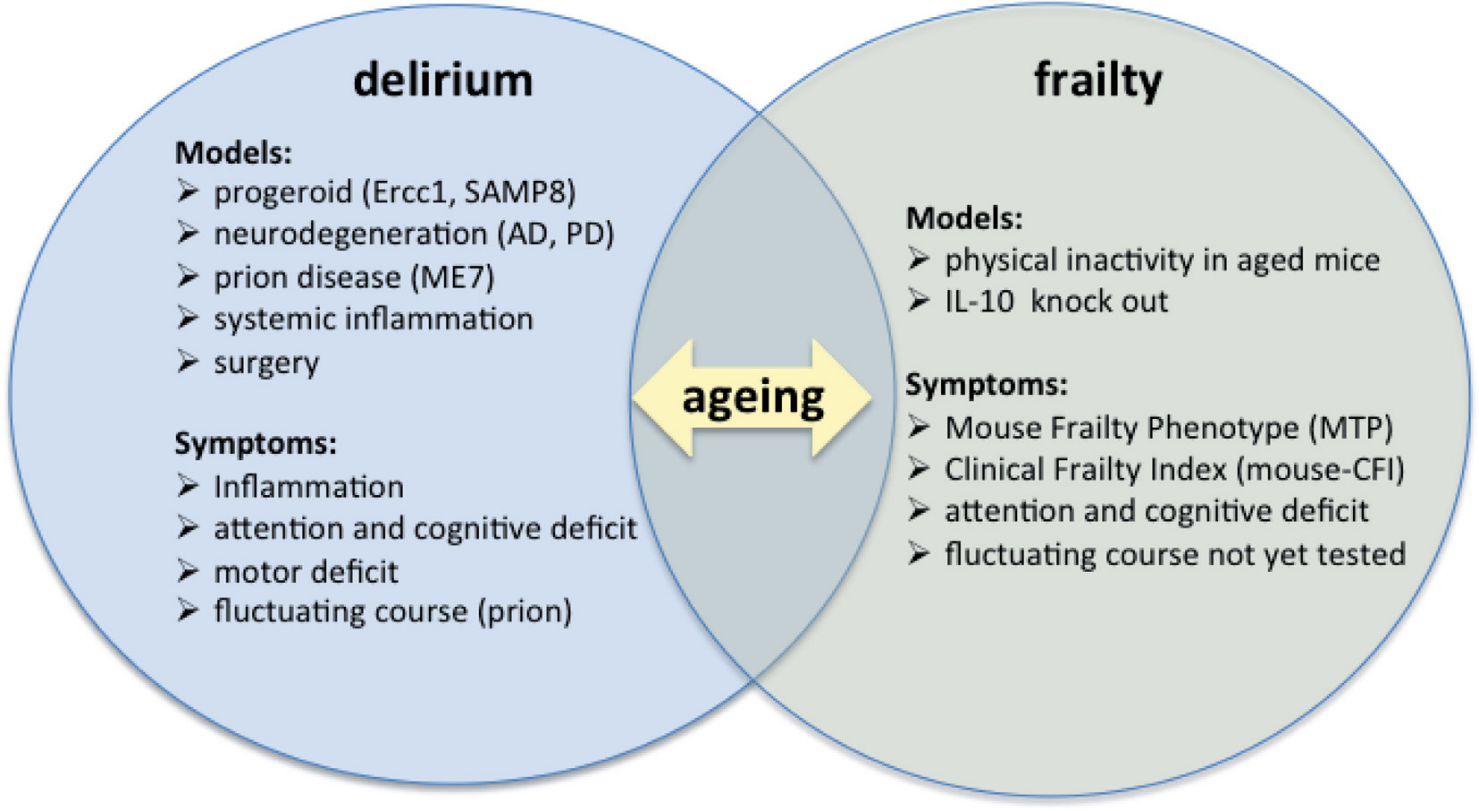
Preclinical models of frailty and delirium. Current studies on preclinical models of delirium and frailty must be put in the context of aging, as shown by the intersection of the two diagrams.

Preclinical animal models may be of particular importance for the study of both frailty and delirium, given the absence of neuropathological studies in humans on these two conditions (Figure 1).

Animal models for delirium are substantially based on the neuroinflammatory hypothesis of delirium. A recent review by Hoogland et al. discuss the studies with animal experiments related to the effects of systemic inflammation on the microglial and inflammatory response in the brain (85). The authors identified 51 studies of which the majority was performed in mice (*n* = 30) or in rats (*n* = 19). Despite heterogeneity in the outcomes measures and in the methods used to assess microglial activation, these studies clearly showed that peripheral inflammatory stimuli can cause microglial activation. The authors also observed distinct differences in microglial activation between systemic stimulation with (supranatural doses) LPS and live or heat-killed bacteria (85). Another systematic review included not only studies with inflammatory challenge but also studies based on the effect of surgery (86). The effect of acute administration of bacterial endotoxin was reported in 29 comparative studies on normal animals (24 LPS and 5 *Escherichia coli* bacterial), 3 studies on progeroid model (Ercc1 mutant mouse deficient in DNA repair and the SAMP8 mouse, characterized by overproduction of amyloid precursor protein and oxidative damage), and 14 studies on disease models related with delirium (ME7 prion disease mice, Tg2576, 3_Tg, and APPswe Tg Alzheimer’s mice, Parkinson disease, basal forebrain cholinergic lesions) (87–89). Furthermore, the effects of different surgery procedures, such as clamping of the upper mesenteric artery, hepatectomy, laparotomy, splenectomy, and appendectomy, reported in 13 comparative studies were also included (10 in mice; 3 in rats) (90). It was found that, in a comparison of adult with young animals, acute peripheral challenges in old animals induce a highest inflammatory response. In particular, studies found that ageing was associated with (a) higher circulating (IL6, TNF-α, and IL10) and brain (IL1b, IL6, TNF-α) cytokines levels or transcripts, (b) increased activated microglia cells and astrocytes, and (c) sickness behavior and reduced cognitive skills with reduced performance at different tests including those evaluating to anxiety, attention or cognition, or activity (fear conditioning, water maze, novel object recognition, attentional set-shifting, social exploratory, general activity, or locomotor test). Considering the effect of surgery, the majority of studies reported an increase of brain proinflammatory cytokines (IL-1b, TNF-a, IL-6). Activation of microglia or astrocytes, increase of neuronal apoptosis, and loss of neuronal dendritic spine were also reported in some studies, confirming a tight link between primed microglial cells and neuronal plasticity (91). It should also be considered that neurodegeneration (or the presence of progeroid genetic defects) may anticipate the exaggerated inflammatory response that it is associated with a more rapid cognitive decline and disease progression. Administration of low doses of LPS (100 μg/kg) in ME7 animals, for example, induced transient working memory deficits with increased and prolonged transcription of inflammatory mediators in the brain. Results of these studies indicate that preexisting synaptic loss and microglial priming are predisposing factors for acute cognitive impairments provoked by systemic inflammation. In the same animal model, the peripheral administration of a single proinflammatory cytokine, like TNF-α in pre-symptomatic phase, is able to produce an exaggerated sickness behavior response but not neuronal death, synaptic loss, or hyperphosphorylation of tau. In a recent study, a fluctuating course of cognitive dysfunction was also reported in ME7 mice injected with 0.1 mg/kg LPS. LPS precipitated severe and fluctuating cognitive deficits in 16-week ME7 with a lower incidence or no deficits in 12-week ME7 and controls, respectively. Fluctuating impairments were associated with progressive thalamic synaptic loss and axonal pathology (14, 92).

Another relevant finding for delirium was obtained in a mouse model of lesioned basal forebrain cholinergic system, based on the administration of ribosomal toxin saporin linked to the p75 neurotrophin receptor. In this model, cognitive deficits induced by systemic LPS are restored by donepezil. However, in this model no signs of increased brain inflammation were detected, suggesting that factor other than primed microglial cells may be involved in the development of cognitive dysfunction and that neuronal vulnerability may represent predisposing factor to peripheral inflammation associated cognitive impairment (87).

Various tools for the assessment of frailty have been developed in mice, based on the assessment tools used in humans (93). Parks et al. proposed a preclinical frailty Index scale based on the assessment of deficits in activity skills, body composition, metabolic status, and vascular system (94). Whitehead and colleagues developed a mouse Clinical Frailty Index (mouse-CFI) assessment tool based on a 31 different impairments and deficits (95) and Liu et al. developed the Mouse Frailty Phenotype (MTP) scale, including grip strength, speed in walking, physical activity, and endurance (96). These tools have demonstrated to be consistent in frailty assessment in that (a) their scores increase in severity with aging in both male and female; (b) the increase in score severity which is observed with aging is similar to that observed in humans; and (c) the age-associated changes in myocytes are more prominent in animals with elevated frailty scores than in others. In a recent study (97), the mouse-MTP scale and the mouse-CFI were compared in a group of mice aged 23–24 months. Using the first tool, none of the mice was classified as frail. On the contrary, the second tool classified 16.6% of mice as frail. As indicated by the authors, similar difference in estimating the true incidence of frailty can be found when the frailty phenotype model and the frailty index tools are compared in humans: indeed, 6–16% of older adults (70–85 years old) are defined as frail with the phenotype-model tool and 22–32% with the FI (65 years and older) (98, 99). Despite the different sensitivity between the tools, however, both are able to detect frailty at preclinical level (93). Based on the known association between physical activity and frailty, Gomez-Cabrera et al. proposed physical inactivity as a mouse model of frailty (100). They adapted to animals the frailty phenotype developed for human and defined a score (the Valentia score for frailty evaluation) to be used in mice. Scores were calculated on the basis of five different components, such as weight loss (change in body weight), weakness (grip strength), poor endurance and slowness (incremental treadmill test), and low activity level (motor coordination), and were expressed with frailty scores similar to these defined for human being. Sedentary and wheel runner animals were compared in longitudinal study until the age of 28 months. Results of the study indicate that sedentary animals become frail as they get older whereas lifelong spontaneous exercise signicantly retards the onset of frailty.

Another animal model for frailty is the IL-10 (tm/tm) mouse developed by Walston (101). The lack of the anti-inflammatory cytokine interleukin-10 (IL-10) makes this animal more susceptible to the activation of inflammatory pathway activation. With aging, this mouse shows higher than normal levels of circulating IL-6, reduced muscular strength, impaired skeletal muscle ATP kinetics and cardio-vascular functions, and increased expression of gene associated with the regulation mitochondrial function and apoptosis (102).

## Conclusion and Future Directions

There is initial evidence that frailty and delirium might share common pathophysiological links and are strictly interrelated from a clinical perspective. Altered inflammatory status is clearly involved in the pathophysiology of both frailty and delirium. Muscle and adipocytes may be a source of inflammatory stimulus (through endocrine secretion of pro-inflammatory adipokines) and also a target of the negative effects (i.e., induction of catabolic, apoptosis, autophagic muscular pathways) (81, 82). The inflammatory markers produced at the muscle and adipocytes level, on one side, may enter the brain through neural and/or humoral pathways, priming microglia and other neuronal cells of the brain that react with overexpression of cytokines. The primed microglia are able, under these conditions, to promote neuronal dysfunction leading to cognitive and behavioral symptoms of delirium (85).

Another pathophysiological link may be at vascular level. Pericytes are spatially isolated contractile cells on capillaries and venules throughout the body, which are designated to control cerebral blood flow physiologically, and to limit blood flow after ischemia. In skeletal muscle, pericytes are located at the interstitium, where they can express typical markers associated with capillaries. Pericytes have also adipogenic and myogenic properties, contributing to muscle fat generation and lipotoxicity. It could be hypothesized that an exaggerated activation of these cells leads to increased inflammatory response and nitrosative stress in the muscle, thus contributing to sarcopenia, a key feature of frailty. At the brain level, pericytes may contribute to enhance the inflammatory response under specific circumstances, which may represent both a predisposing and a precipitating factor for delirium occurrence in frail subjects. Pericyte alterations may also be responsible for increased permeability of the blood brain barrier microvascular endothelium, which in turn may lead to an overexpression of inflammatory markers in the brain and overactivation of microglial cells (103). Indeed, it has been demonstrated an upregulation of proteins in the cerebrospinal fluid of delirious patients within clusters related to inflammation, protease inhibitors, chromogranins/secretogranins and apolipoproteins, triggered by infections, metabolic problems and adverse drug reactions (104). Increased oxidative stress and neuroendocrine abnormalities may also occur at both levels (i.e., at the body and brain levels), igniting a chain of reactions that include overexpression of cytokines and other inflammatory markers.

The review also suggests that the complex relationship between frailty and delirium should be thought as a dynamic and continuous cross-talk between the body and the brain. Future studies should therefore try to identify biomarkers specific for body cells and brain in frail individuals with delirium. An excellent example of the cross-talk between muscle and brain should be represented by the Brain-derived neurotrophic factor (BDNF). BDNF is strongly expressed in the brain (105), where it regulates neuronal development, synaptic plasticity, and influence memory (106) but it is also expressed in skeletal muscle, where it contributes to fat oxidation and modulates myogenesis inducing satellite cell activation and skeletal muscle regeneration (107, 108).

From a clinical perspective, frailty may be considered a risk factor for delirium, although full evidence is still lacking, and delirious individuals may be regarded by default as frail individuals. Moreover, delirium may be viewed, in some cases (e.g., when it persists for long time) as a precipitating factor for worsening frailty. Specific attention will therefore be paid by clinicians both on frailty assessment, since it may allow to anticipate delirium occurrence and to the systematic screening of delirium since it may help identifying individuals at risk of subsequent worsen of frailty.

With regard to animal models, future research is needed to identify a panel of biomarkers that should be relevant both in humans with delirium and in mouse models of frailty, specifically challenged with triggers causing delirium, in order to explore new pathophysiological pathways.

## Author Contributions

All authors drafted and reviewed the manuscript.

## Conflict of Interest Statement

The authors declare that the research was conducted in the absence of any commercial or financial relationships that could be construed as a potential conflict of interest.
